# Impact of Subclinical Hearing Loss on Quality of Life Among Industrial Workers: A Cross-Sectional Analysis Utilizing the SF-36 Questionnaire

**DOI:** 10.7759/cureus.87575

**Published:** 2025-07-09

**Authors:** Christos A Karaiskos, Georgios Rachiotis, Effie Simou, Georgios Dounias

**Affiliations:** 1 Public Health Policy, University of West Attica, Athens, GRC; 2 Epidemiology and Occupational Hygiene, University of Thessaly, Larissa, GRC

**Keywords:** industrial health, noise exposure, quality of life (qol), sf36, hearing loss

## Abstract

Background: Occupational noise exposure is a leading cause of hearing impairment, which can affect both physical and psychological well-being. While most research focuses on advanced hearing loss, the impact of mild auditory deficits remains underexplored.

Objective: The study aims to investigate the relationship between varying degrees of hearing loss and health-related quality of life (HRQoL) among industrial workers.

Methods: In this cross-sectional study, 345 full-time workers from three pharmaceutical factories in Attica, Greece, exposed to noise levels >85 dB, were categorized into three groups based on audiometric data: normal hearing (n = 195), mild hearing loss (n = 78), and evident hearing loss (n = 72). HRQoL was assessed using the SF-36 questionnaire. Statistical analyses included the Mann-Whitney U test and Spearman correlation test (significance set at p < 0.05).

Results: Compared to workers with normal hearing, those with mild hearing loss scored significantly lower across all SF-36 dimensions (e.g., physical functioning: 91.5 vs. 95.8, p < 0.001; mental health: 84.8 vs. 94.3, p < 0.001). Even with preserved speech-frequency hearing, the mild group exhibited reduced emotional and social functioning. No significant QoL differences were observed by gender or education, except for bodily pain (p = 0.004). Age correlated negatively with physical functioning only in the group with evident hearing loss (r = -0.27, p = 0.02).

Conclusion: Mild hearing loss can substantially impact the subjective QoL of workers, even in its early stages. Occupational health strategies should include psychosocial screening and counseling, as well as routine audiological monitoring.

## Introduction

Hearing health is a critical factor for general well-being and quality of life (QoL), especially in occupational settings with high noise exposure. Occupational noise exposure has been consistently linked to an increased risk of developing hearing loss, which is one of the most common occupational illnesses worldwide [1]. Beyond objective hearing impairment, auditory burden affects multiple aspects of subjective health, such as emotional balance, social participation, and the perception of functional adequacy [2-6]. QoL, as measured by multidimensional tools such as the SF-36, reflects the physical, psychological, and social dimensions of functioning [7]. Prior studies have shown that hearing loss is associated with increased risk of depression, social isolation, reduced work performance, and even elevated morbidity in older populations [8]. However, most existing literature focuses on severe hearing loss or elderly adults, overlooking milder forms of hearing impairment, particularly in workers chronically exposed to industrial noise. This study aims to address this gap by examining the relationship between even mild auditory burden and the subjective QoL among pharmaceutical industry workers in Greece.

## Materials and methods

This cross-sectional study included all industrial employees from three major pharmaceutical companies in Attica, Greece, between July 2020 and March 2023. Inclusion criteria were (a) full-time employment and (b) noise exposure >85 dB according to health and safety department measurements, in line with national regulations. Exclusion criteria were (a) office-based employees or personnel whose regular work environment consistently measured noise levels below 85 dB, (b) individuals who declined to participate or did not provide informed consent, and (c) workers with pre-existing medical conditions unrelated to noise exposure that could significantly confound audiological or QoL assessments (e.g., severe neurological disorders, diagnosed psychiatric conditions, or acute ear infections at the time of examination). This was assessed via a brief medical history questionnaire administered by the occupational physician. Occupational roles included drug production technicians, packagers, maintenance staff, and cleaners. Employees worked rotating morning and afternoon shifts, with no night shifts.

The study employed a complete enumeration (census) approach, meaning all workers meeting the inclusion criteria within the selected factories during the study period were invited to participate. No random sampling was performed. Out of 510 total workers across the three facilities, 345 met the inclusion criteria and participated in the study. The remaining 165 workers either did not meet specific inclusion criteria (e.g., office-based, noise levels below 85 dB) or declined to participate after receiving comprehensive information about the study.

This cohort was subsequently categorized into three groups based on their most recent audiometric data, collected as part of routine occupational health surveillance: normal hearing (n = 195), mild hearing loss (n = 78), and evident hearing loss (n = 72). Mild hearing loss was defined as normal hearing at speech frequencies (500-4000 Hz), but with a loss of more than 25 dB at 4000 Hz or 8000 Hz. Evident hearing loss was defined as >25 dB average loss at speech frequencies. These audiometric criteria for categorizing hearing loss were based on widely accepted audiological practices and World Health Organization (WHO) guidelines, which define normal hearing thresholds as up to 25 dB of hearing loss across all tested frequencies [9]. This classification acknowledges that while mild hearing loss, particularly at higher frequencies (e.g., 6000 Hz, 8000 Hz), may not immediately impact speech understanding in quiet environments, it signifies an early stage of auditory deficit, often associated with noise exposure. Evident hearing loss implies more significant impairment, including frequencies crucial for speech perception.

Subjective health-related quality of life (HRQoL) was assessed using the SF-36 version 1 questionnaire. The SF-36 version 1 is available for public use and does not require a license for use. Statistical analyses were performed using IBM SPSS Statistics for Windows, Version 29 (Released 2023; IBM Corp., Armonk, New York) and included Mann-Whitney U and Spearman correlation tests (significance set at p<0.05). Ethics approval was granted by the Research Ethics Committee of the University of West Attica (Ref. 5736/21.07.2021). This study was based on a complete enumeration of all eligible workers in the selected sites. As no sampling was performed and the design was demographic in nature, a priori power analysis was not required.

## Results

Group differences

Table 1 compares the groups with evident and mild hearing loss. The mild hearing loss group scored significantly higher in physical role (p = .038), emotional role (p = .046), and social functioning (p = .011), indicating better QoL. Table 2 shows that the mild hearing loss group had significantly lower scores than the normal hearing group across all SF-36 domains (p < .001), despite having normal hearing at speech frequencies. This implies that QoL impairment may be linked not only to communication difficulties but also to subjective or psychosocial factors, such as health-related anxiety, feelings of vulnerability, or early awareness of physiological deterioration.

**Table 1:** Comparison of SF-36 dimensions between workers with evident and mild hearing loss

SF-36 Dimension	Evident Hearing Loss (n=72)	Mild Hearing Loss (n=78)	z-score (p-value)
Physical Functioning	87.15	91.54	-0.856 (.195)
Role Physical	82.64	88.78	1.776 (.038)
Role Emotional	80.57	87.19	1.687 (.046)
Vitality	79.04	91.35	1.061 (.145)
Mental Health	83.06	84.83	0.809 (.209)
Social Functioning	84.86	90.23	2.291 (.011)
Bodily Pain	88.34	89.26	0.685 (.248)
General Health	76.44	81.41	2,033 (.211)

**Table 2:** Comparison of SF-36 dimensions between workers with mild hearing loss and normal hearing

SF-36 Dimension	Mild Hearing Loss (n=78)	Normal Hearing (n=195)	z-score (p-value)
Physical Functioning	91.54	95.84	5.676 (< .001)
Role Physical	88.78	95.73	3.778 (< .001)
Role Emotional	87.19	95.84	3.276 (< .001)
Vitality	91.35	92.95	6.145 (< .001)
Mental Health	84.83	94.28	6.396 (< .001)
Social Functioning	90.23	96.51	4.492 (< .001)
Bodily Pain	89.26	94.95	6.435 (< .001)
General Health	81.41	92.29	8.061 (< .001)

Confounding factors

Gender

No significant gender differences were observed in any SF-36 domain (p > 0.05), as shown in Tables 3, 4.

**Table 3:** Dimensions by gender among workers with evident hearing loss

SF-36 Dimension	Men (n=58)	Women (n=14)	z-score (p-value)
Physical Functioning	87.5	85.71	-0.1383 (0.8893)
Role Physical	82.5	83.93	0.3656 (0.7146)
Role Emotional	80.17	82.86	0.2783 (0.7809)
Vitality	78.28	79.29	-0.0705 (0.9438)
Mental Health	83.10	85.71	-0.0705 (0.9438)
Social Functioning	84.48	85.71	0.0607 (0.9516)
Bodily Pain	87.93	89.29	-0.0705 (0.9438)
General Health	75.86	75.00	0.3275 (0.7435)

**Table 4:** Dimensions by gender among workers with mild hearing loss

SF-36 Dimension	Men (n=48)	Women (n=30)	z-score (p-value)
Physical Functioning	91.98	90.83	0.0205 (0.9840)
Role Physical	88.02	90.00	-0.7600 (0.4473)
Role Emotional	84.05	92.23	-1.5046 (0.1336)
Vitality	98.23	80.33	1.0014 (0.3173)
Mental Health	85.60	83.60	0.6625 (0.5093)
Social Functioning	91.15	88.75	0.9346 (0.3524)
Bodily Pain	88.70	90.16	-0.5495 (0.5823)
General Health	80.83	82.33	-0.2568 (0.7949)

Education

No significant differences were found by education level, except for the pain domain in the mild group, where higher-educated participants reported significantly better scores (p = 0.004), as presented in Tables 5, 6.

**Table 5:** Dimensions by education level among workers with evident hearing loss

SF-36 Dimension	Secondary Education (n=58)	Tertiary Education (n=14)	z-score (p-value)
Physical Functioning	86.47	90.00	-0.8822 (0.3789)
Role Physical	82.50	83.21	0.2917 (0.7718)
Role Emotional	79.32	85.73	-0.6758 (0.4965)
Vitality	80.02	75.00	1.1240 (0.2627)
Mental Health	83.79	80.00	1.3161 (0.1868)
Social Functioning	84.70	85.71	0.1067 (0.9124)
Bodily Pain	87.76	90.62	-1.1240 (0.2627)
General Health	75.67	79.64	-0.6403 (0.5222)

**Table 6:** SF-36 dimensions by education level among workers with mild hearing loss

SF-36 Dimension	Secondary Education (n=52)	Tertiary Education (n=26)	z-score (p-value)
Physical Functioning	91.15	92.31	-0.4611 (0.6455)
Role Physical	87.02	92.31	-1.4680 (0.1416)
Role Emotional	86.56	88.47	-0.1325 (0.8966)
Vitality	81.73	82.88	-0.5406 (0.5892)
Mental Health	85.33	83.85	0.4081 (0.6818)
Social Functioning	89.43	91.83	-0.9290 (0.3524)
Bodily Pain	87.55	92.69	-2.8513 (0.0044)
General Health	80.87	82.50	-0.7138 (0.2389)

Age

Tables 7, 8 revealed a negative correlation between age and physical functioning (r = -0.27, p = 0.02) in the evident hearing loss group; no significant associations were observed in the mild group.

**Table 7:** Spearman correlations between SF-36 dimensions and age (evident hearing loss)

SF-36 Dimension	r (Spearman)	p-value
Physical Functioning	-0.2748	0.0195
Role Physical	-0.1193	0.3181
Role Emotional	-0.0521	0.6637
Vitality	-0.0983	0.4113
Mental Health	-0.0688	0.5660
Social Functioning	-0.1257	0.2927
Bodily Pain	-0.0204	0.8653
General Health	-0.0804	0.5022

**Table 8:** Spearman correlations between SF-36 dimensions and age (mild hearing loss)

SF-36 Dimension	r (Spearman)	p-value
Physical Functioning	-0.0019	0.9871
Role Physical	-0.0827	0.4718
Role Emotional	0.0674	0.5580
Vitality	0.0674	0.5580
Mental Health	0.1060	0.3555
Social Functioning	-0.0144	0.9006
Bodily Pain	-0.0322	0.7797
General Health	-0.0414	0.7190

## Discussion

The present study demonstrates that even mild degrees of hearing loss, despite normal thresholds at speech frequencies, can negatively affect multiple dimensions of HRQoL in industrial workers. This finding aligns with growing evidence that subclinical auditory deficits may contribute to psychosocial strain, emotional burden, and perceived vulnerability, particularly in noise-exposed occupational environments [10,11]. Participants with mild hearing loss exhibited significantly lower SF-36 scores across all domains compared to those with normal hearing. These reductions were not limited to physical functioning but extended to emotional role, social functioning, and mental health, underscoring the broader impact of early-stage auditory dysfunction. The fact that such impairments occur despite preserved speech-frequency hearing suggests that standard audiometry may underestimate the true functional burden of noise-induced cochlear damage, especially at higher frequencies. This study contributes to the literature by focusing on a relatively underexplored population: younger, working adults in a pharmaceutical manufacturing setting, rather than elderly individuals or those with clinically diagnosed conditions.

The absence of significant differences by gender or education level (with the exception of bodily pain) suggests that the detrimental impact of mild hearing loss on QoL may transcend sociodemographic boundaries. The observed inverse correlation between age and physical functioning in the group with evident hearing loss is consistent with existing literature, indicating that age-related hearing decline may exacerbate functional limitations and reduce resilience to workplace stressors. Importantly, these findings have implications for occupational health policy and prevention. Current European and international guidelines (e.g., the European Union Information Agency for Occupational Safety and Health at Work (EU-OSHA) and WHO) emphasize exposure monitoring and the use of personal protective equipment but rarely address the early psychosocial consequences of hearing loss [12].

Our results support the need for a broader approach that includes routine audiometric screening, functional hearing assessments (e.g., speech-in-noise tests), and access to psychological support for affected workers [13]. Recent international findings further support the association between mild hearing loss and reduced QoL, highlighting the beneficial role of hearing aid use in improving HRQoL (mean HUI3 change ≈ +0.11) [14]. Nevertheless, occupational noise exposure remains widespread: CDC/NIOSH (Centers for Disease Control and Prevention/National Institute for Occupational Safety and Health) estimates indicate that approximately 25% of workers are exposed to hazardous noise levels, with less than half consistently using hearing protection [15]. The psychosocial consequences of mild hearing loss include higher rates of depression and social isolation, particularly in low socioeconomic populations, as shown in the England Longitudinal Study of Ageing (2021) [16]. The WHO's "World Report on Hearing" (2021) highlights that hearing loss is significantly underdiagnosed and neglected globally, with major implications for psychosocial health [17]. Recent research also highlights how noise-induced hearing loss in workers diminishes job performance and workplace well-being [18]. Integrating such strategies into workplace health programs could enhance worker well-being, reduce absenteeism, and prevent long-term disability [19]. Moreover, raising employer awareness of the early impact of auditory strain could foster a more supportive and inclusive work environment.

Limitations

Our study has several limitations. Its cross-sectional design, as acknowledged, precludes the establishment of causality between hearing loss and HRQoL dimensions, allowing only for the examination of associations. Furthermore, the use of self-reported assessment tools such as the SF-36 questionnaire introduces potential biases, including social desirability bias and variability in personal health perception. Additionally, the sample consisted exclusively of workers from the pharmaceutical manufacturing sector, which limits the generalizability of the findings to other occupational groups or production environments. While our study focused on occupational noise, we did not collect detailed data on potential unmeasured confounders, such as non-occupational noise exposure (e.g., recreational noise, personal music device use, exposure from daily activities), which could also contribute to an individual's hearing status.

The potential influence of age-related hearing decline, beyond what was observed in the evident hearing loss group, and the possible long-term effects of conditions like COVID-19 on auditory health were also not specifically assessed and could represent additional unmeasured confounders. Information regarding specific medication use by participants, particularly ototoxic medications or those affecting QoL, was not collected and could also potentially confound the results. No data were collected regarding the duration of noise exposure, adherence to hearing protection use, or the pre-existing psychosomatic health status of participants, factors that may confound perceived QoL outcomes.

Finally, hearing ability was assessed solely through audiometry, rather than through functional tests such as speech-in-noise comprehension. Given that perceived hearing often differs from audiometric thresholds, future research should incorporate functional hearing assessments. Future longitudinal studies with more comprehensive data collection on these factors would be valuable to further elucidate the intricate relationship between noise exposure, hearing health, and QoL.

## Conclusions

This study demonstrated a significant association between even mild forms of subclinical hearing loss and a measurable decline across multiple dimensions of HRQoL among industrial workers exposed to high levels of noise. Our findings underscore that the impact of early-stage auditory deficits extends beyond pure communication difficulties, affecting psychological and social well-being. This evidence highlights the critical need for a more comprehensive approach to occupational hearing health. Beyond traditional noise exposure mitigation, prevention strategies should be broadened to include regular, sensitive hearing screenings capable of detecting early changes, alongside the provision of psychosocial support and counseling for affected individuals. Furthermore, fostering greater employer awareness regarding the pervasive consequences of even subclinical hearing loss on worker well-being and productivity is essential. Future longitudinal research is warranted to elucidate causal pathways and to develop and evaluate targeted interventions aimed at mitigating the full spectrum of QoL impairments associated with occupational noise exposure.
